# Supplementary figures and images for: Transcription-replication conflicts as a source of common fragile site instability caused by BMI1-RNF2 deficiency
Source: PLoS Genet. 2020 Mar 6;16(3):e1008524. doi: 10.1371/journal.pgen.1008524 (PMC7080270; doi:10.1371/journal.pgen.1008524)

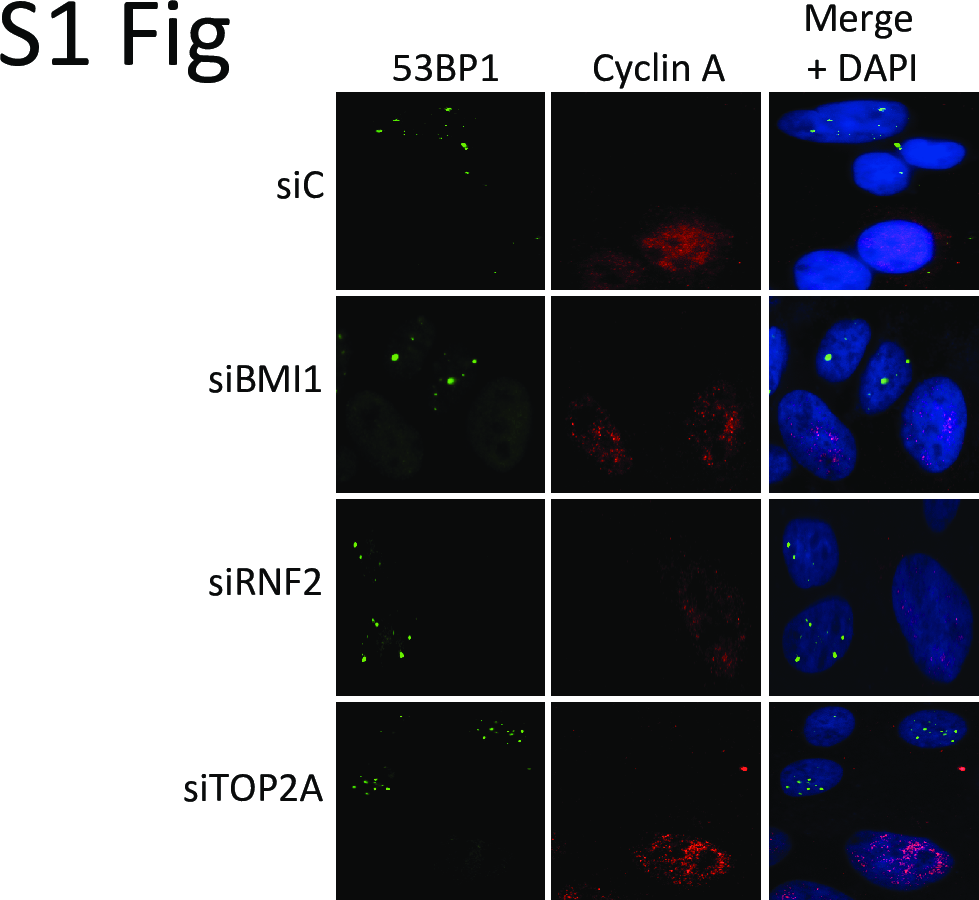

Supplement: S1 Fig — (Quantification is shown in Fig 1A). (TIF) [file pgen.1008524.s001.tif]

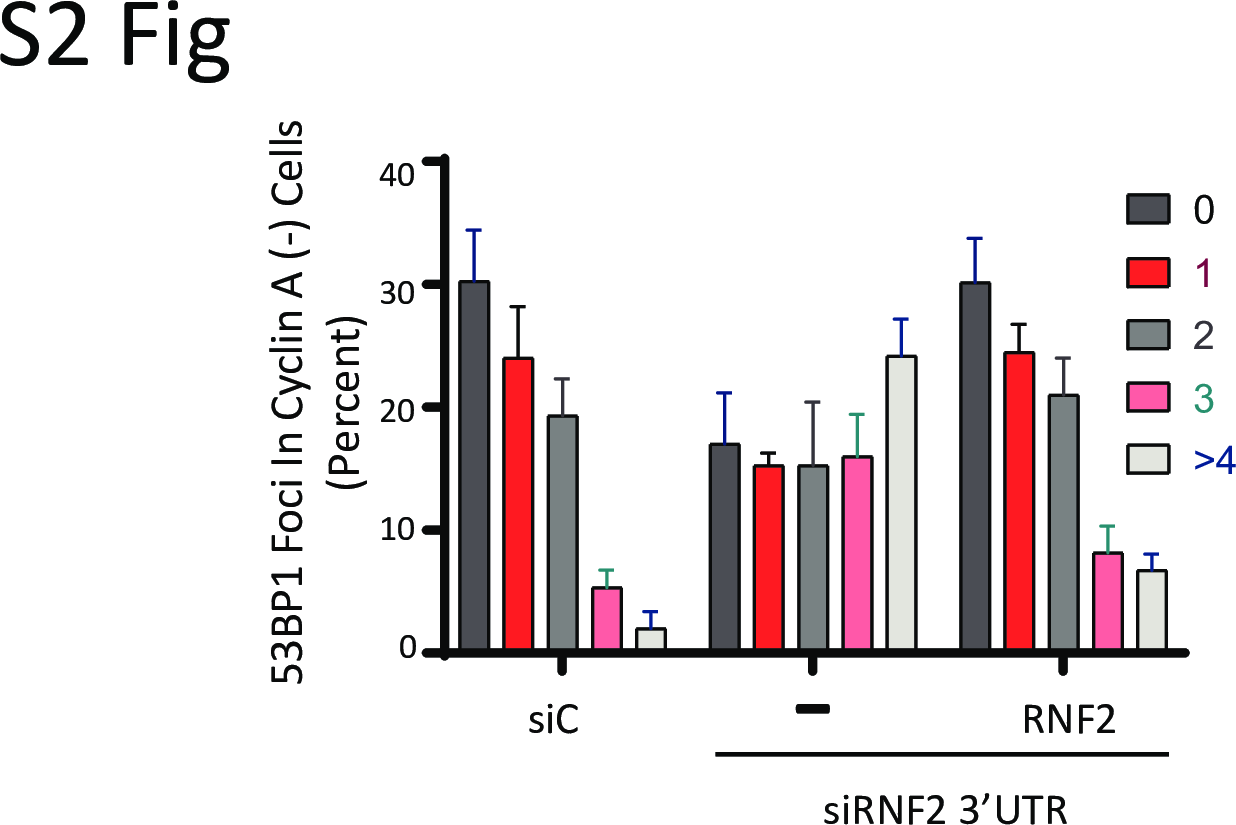

Supplement: S2 Fig — At 72 hours post-transfection, cells were fixed and analyzed for 53BP1 foci (in Cyclin A-negative cells). Assays were done in triplicates (N = 100 for each condition). (TIF) [file pgen.1008524.s002.tif]

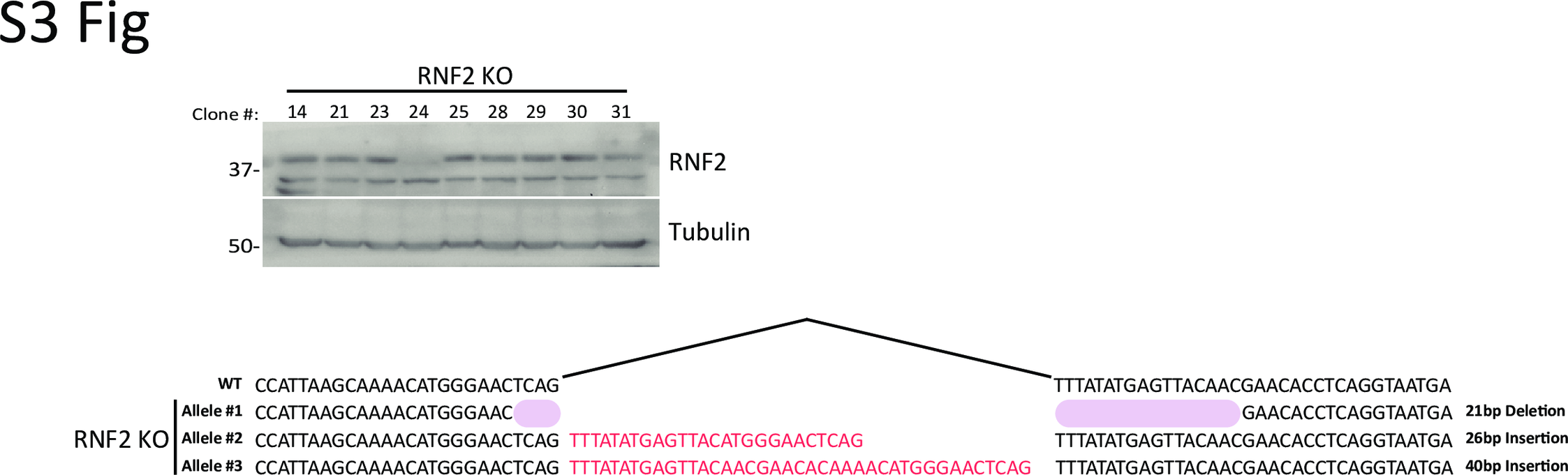

Supplement: S3 Fig — (Top) Western blot screening identifies the clone #24 as true T80 RNF2 CRISPR KO clone. (Bottom) Sequencing analysis of the RNF2 KO clone #24. (TIF) [file pgen.1008524.s003.tif]

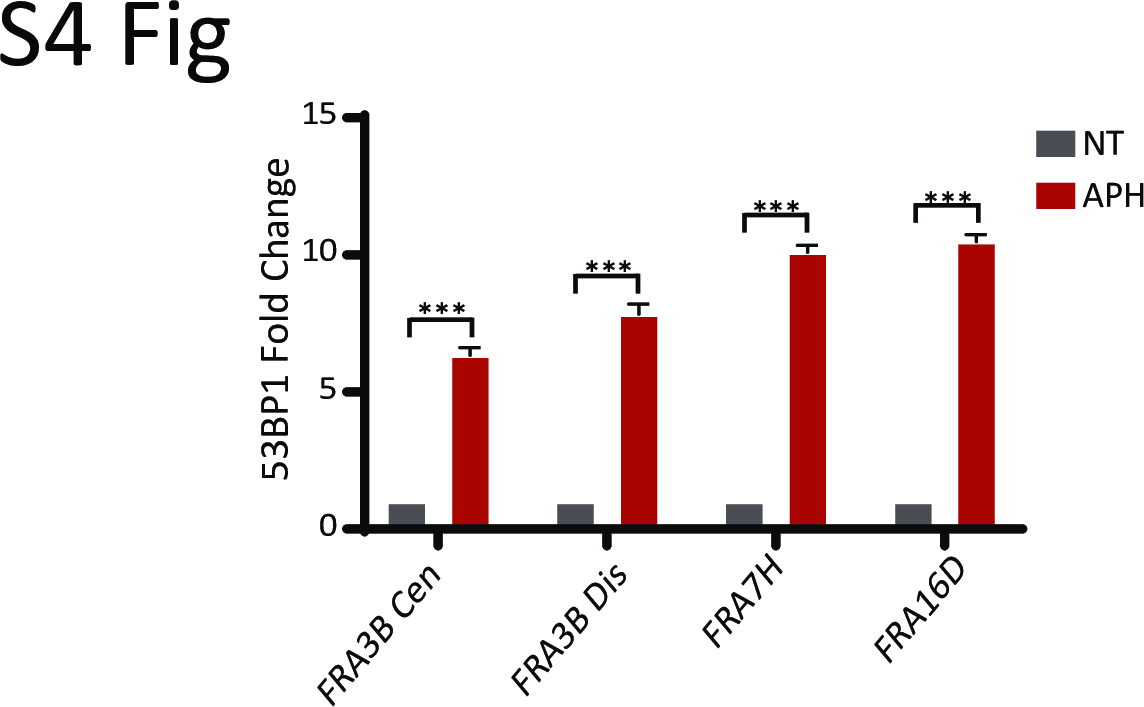

Supplement: S4 Fig — (N = 3 biological replicates; ***P < .0005,**P < .005, *P < .01). (TIF) [file pgen.1008524.s004.tif]

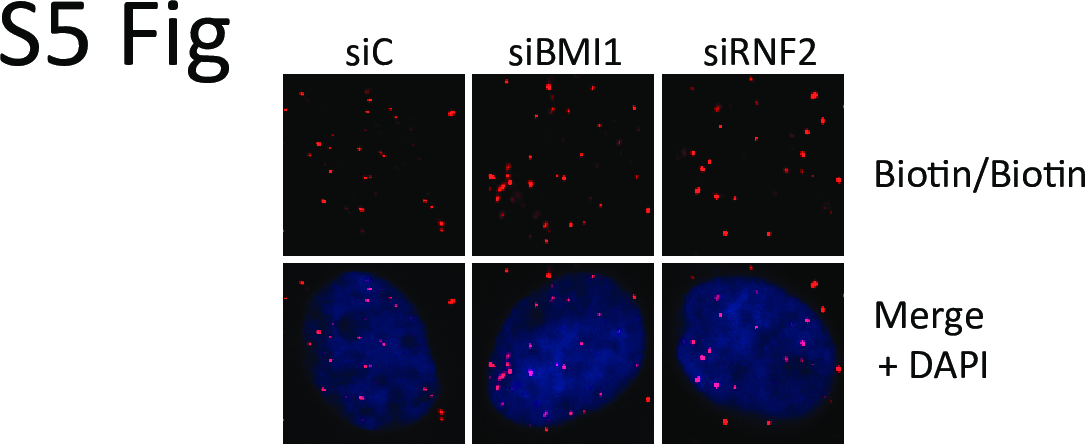

Supplement: S5 Fig — The cells were probed with mouse and rabbit biotin antibodies and used for a PLA reaction to determine if the extent of EdU labeling was equal among the conditions. (N = 3 biological replicates). These cells were set up simultaneously with sample probed for RPA32 and EdU (Fig 2E). (TIF) [file pgen.1008524.s005.tif]

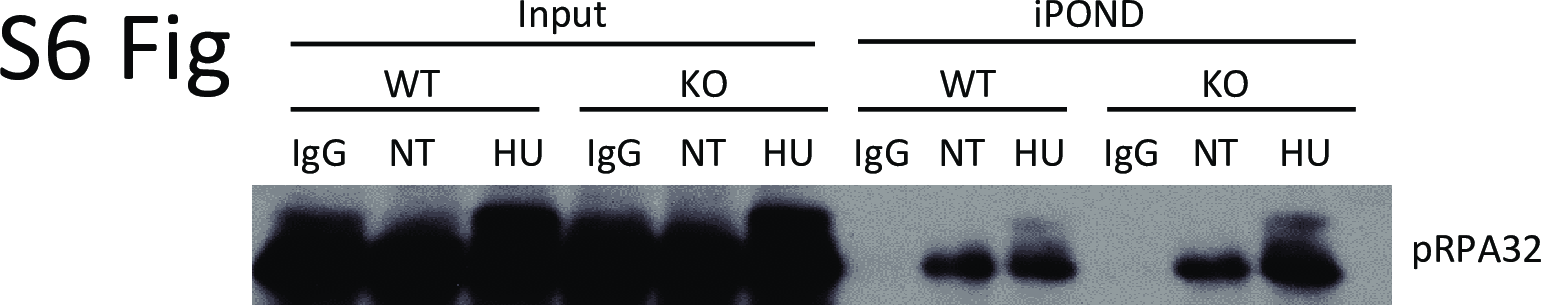

Supplement: S6 Fig — Where indicated cells were treated with 2mM HU for 16 hrs. (TIF) [file pgen.1008524.s006.tif]

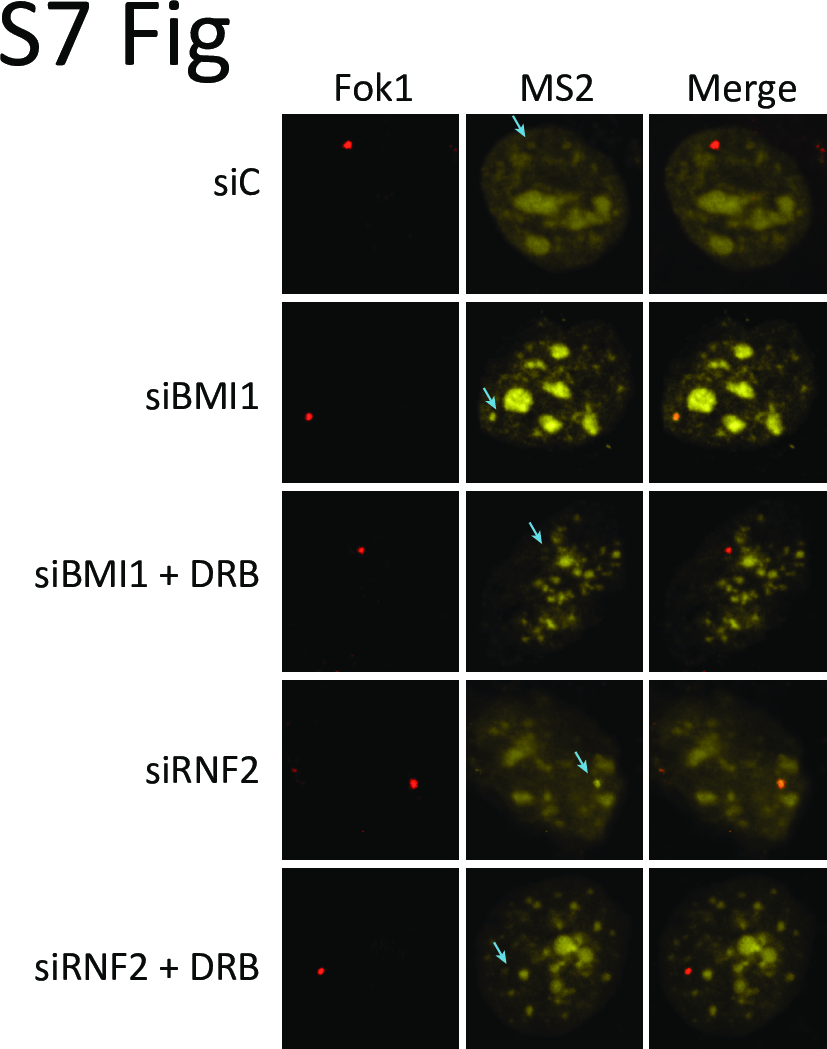

Supplement: S7 Fig — This effect is reversed by treatment with 50uM DRB. (TIF) [file pgen.1008524.s007.tif]

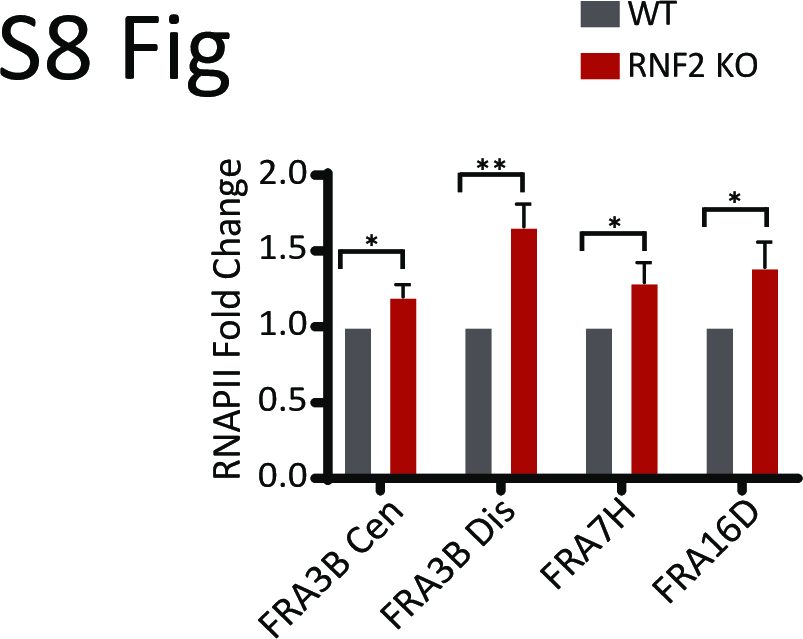

Supplement: S8 Fig — T80 wild type and RNF2 KO cells were IP’ed with anti-Rpb1 (P-Ser2) antibody and the bound DNA was amplified with the indicated primers. (N = 3 biological replicates). (TIF) [file pgen.1008524.s008.tif]

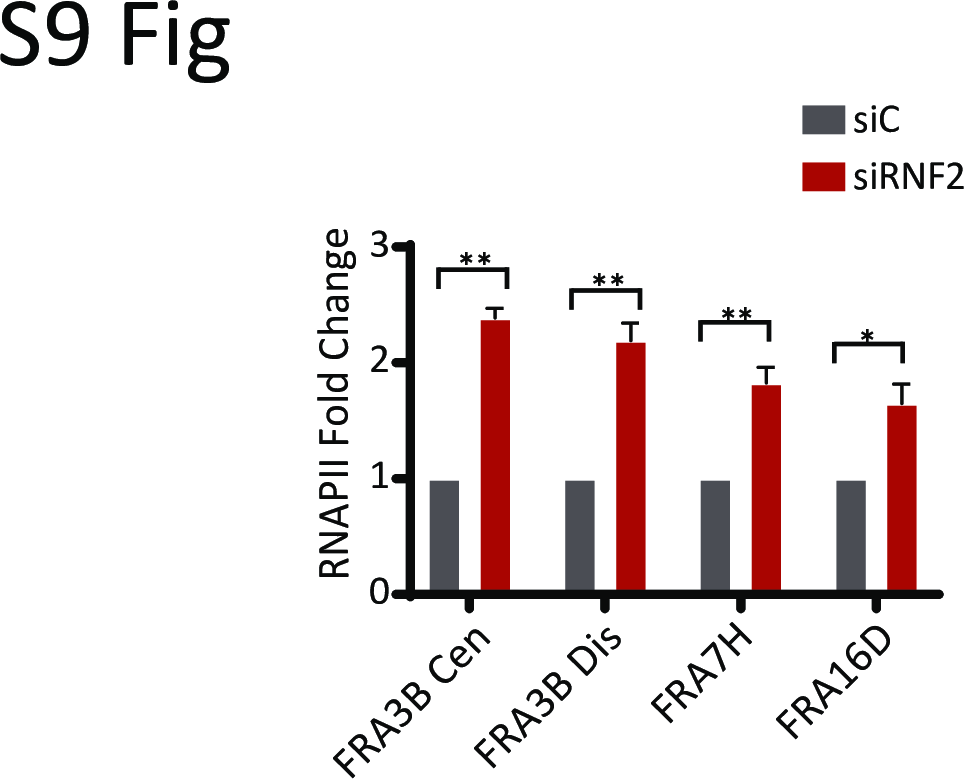

Supplement: S9 Fig — siControl and RNF2-knockdown T80 Cells were IP’ed with anti-Rpb1 (P-Ser2) antibody and the bound DNA was amplified with the indicated primers (N = 3 biological replicates; **P < .005, *P < .01). (TIF) [file pgen.1008524.s009.tif]

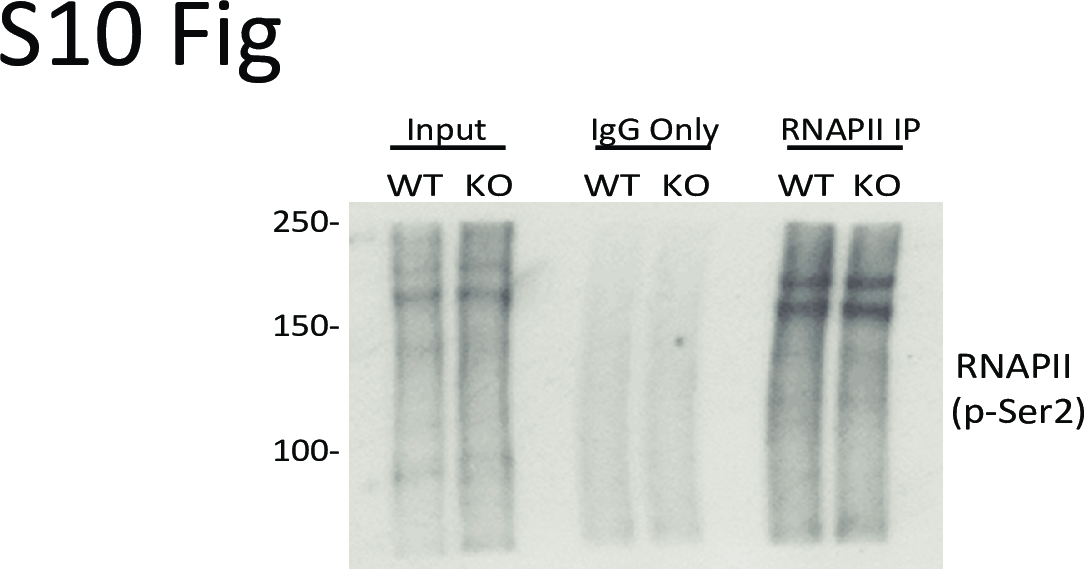

Supplement: S10 Fig — (TIF) [file pgen.1008524.s010.tif]

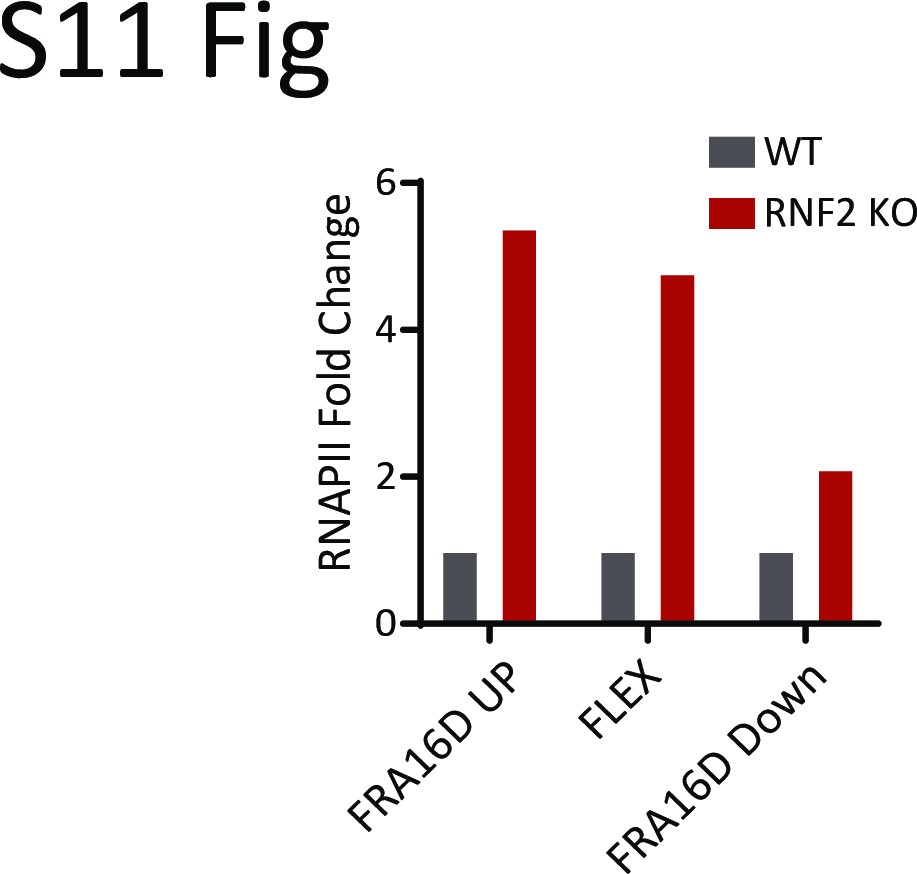

Supplement: S11 Fig — (Amplification with 3 primer sets within the FRA16D region.) (TIF) [file pgen.1008524.s011.tif]

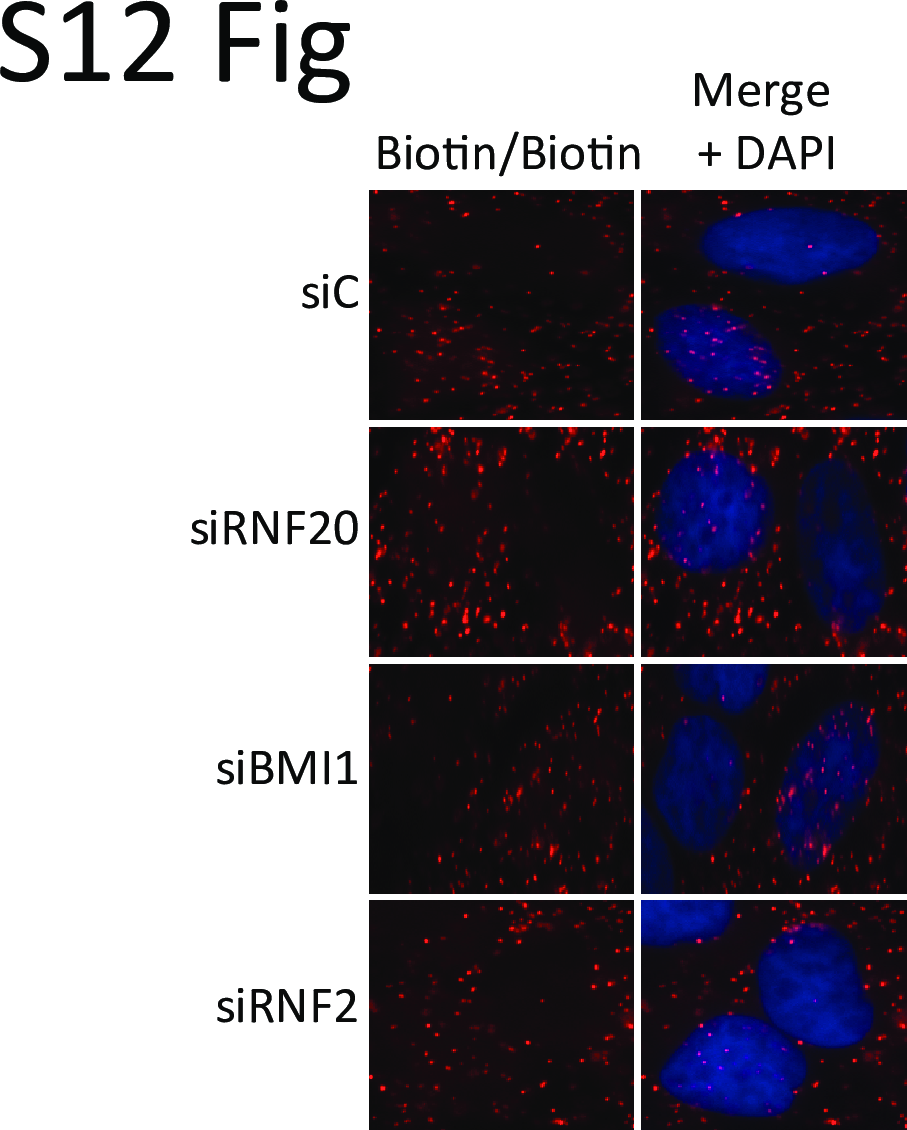

Supplement: S12 Fig — The cells were probed with mouse and rabbit anti-biotin antibodies and used for PLA reactions to determine if the extent of EdU labeling was equal between all conditions. (N = 3 biological replicates). These cells were set up simultaneously with sample probed for Rpb1 and EdU (Fig 3H). (TIF) [file pgen.1008524.s012.tif]

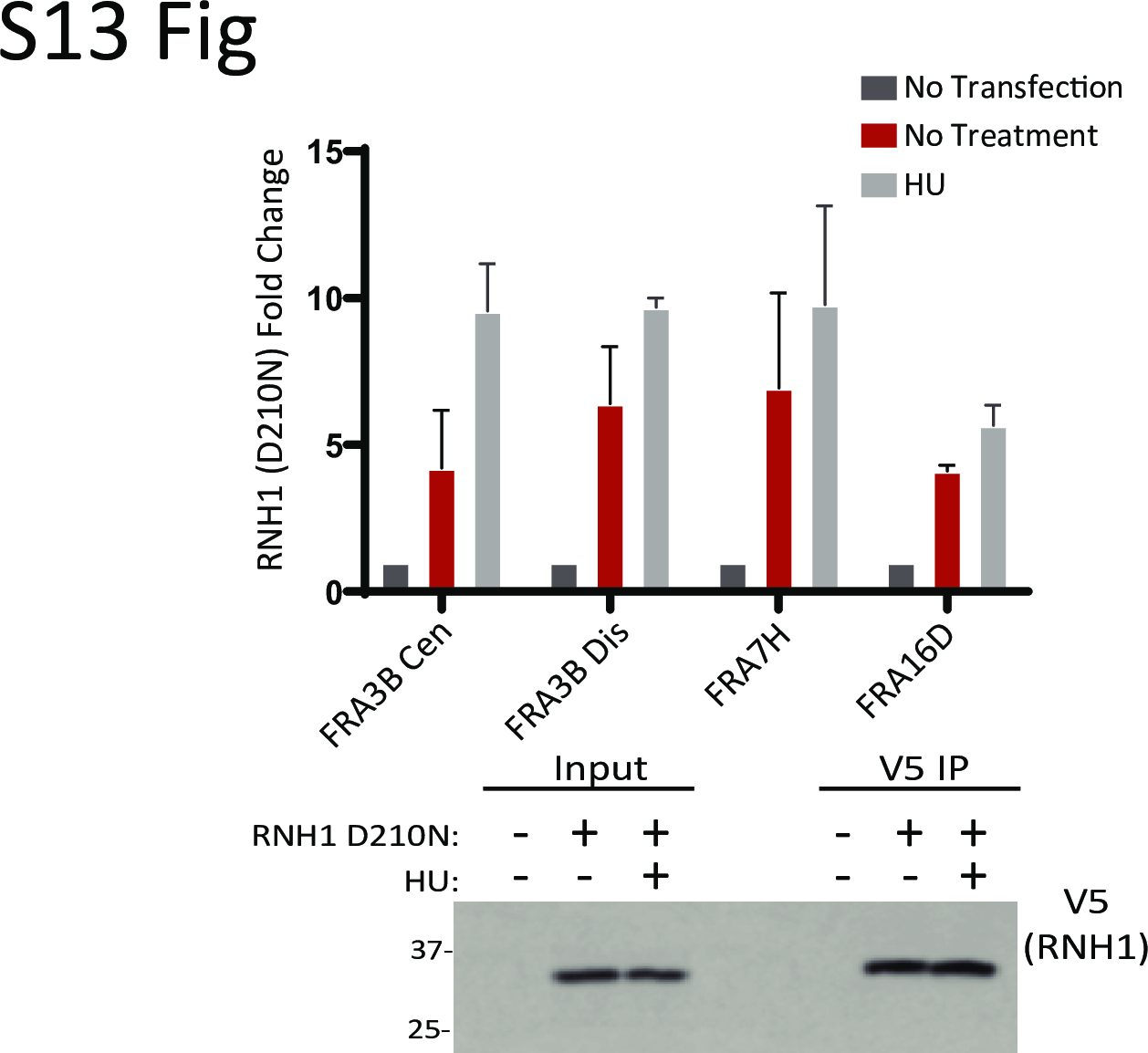

Supplement: S13 Fig — (Top) Quantification of end-point ChIP assay from T80 cells transfected with pyCAG_RNaseH1_ D210N. Cells were subsequently treated with HU (2mM) and IP’ed with anti-V5 antibody (N = 2 biological replicates). (Bottom) Anti-V5 western blot confirming the RNH1 expression and IP efficiency. (TIF) [file pgen.1008524.s013.tif]

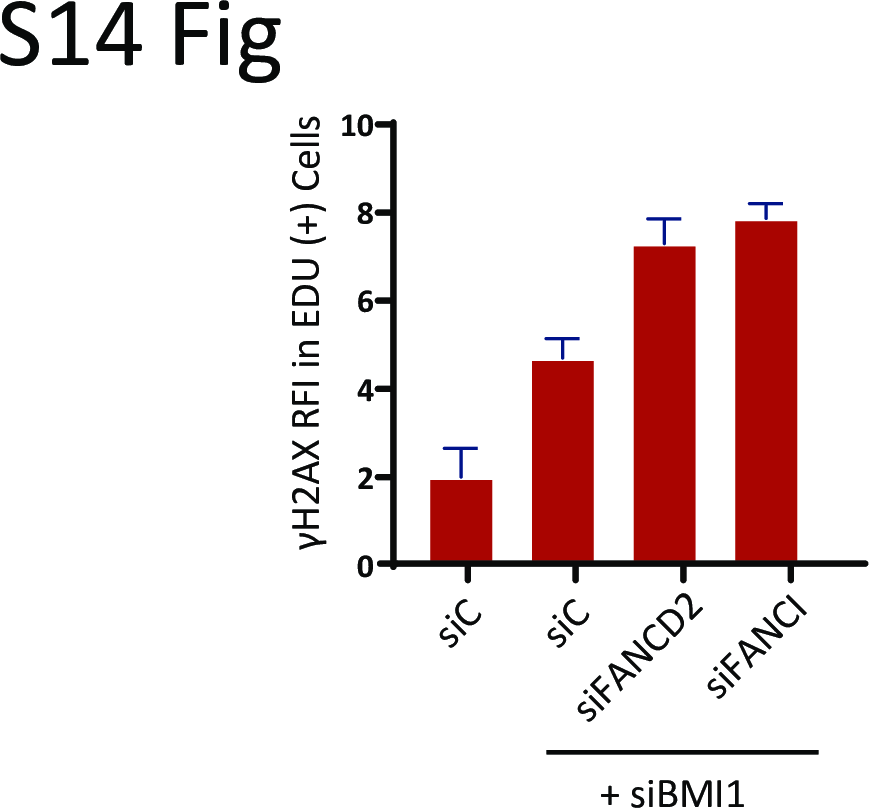

Supplement: S14 Fig — Where indicated, FANCD2 and FANCI were co-depleted by siRNAs. (N = 50 from 3 biological replicates) (TIF) [file pgen.1008524.s014.tif]

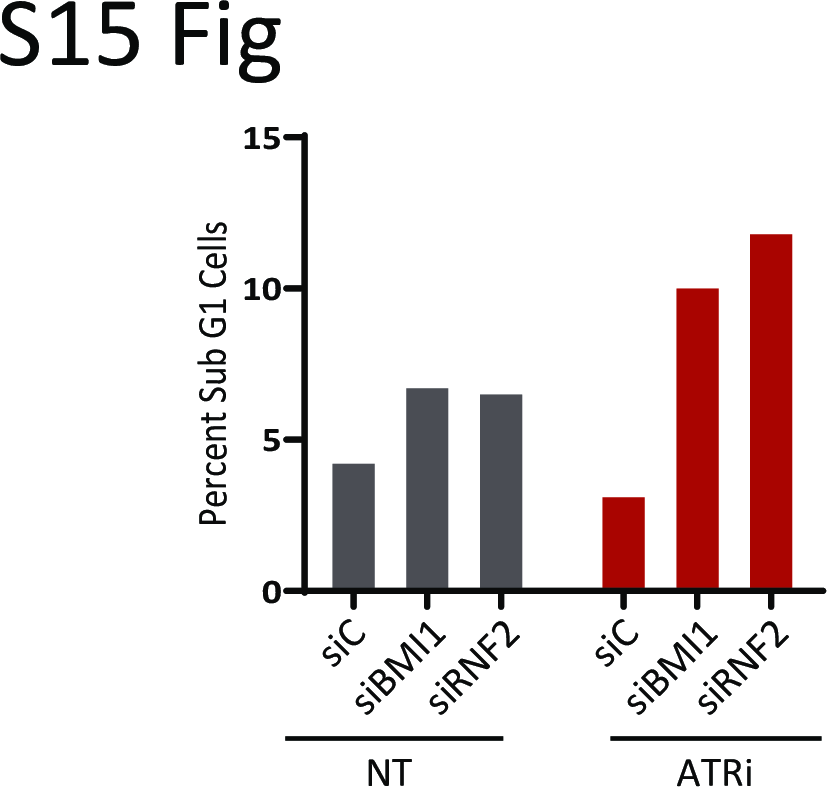

Supplement: S15 Fig — (TIF) [file pgen.1008524.s015.tif]

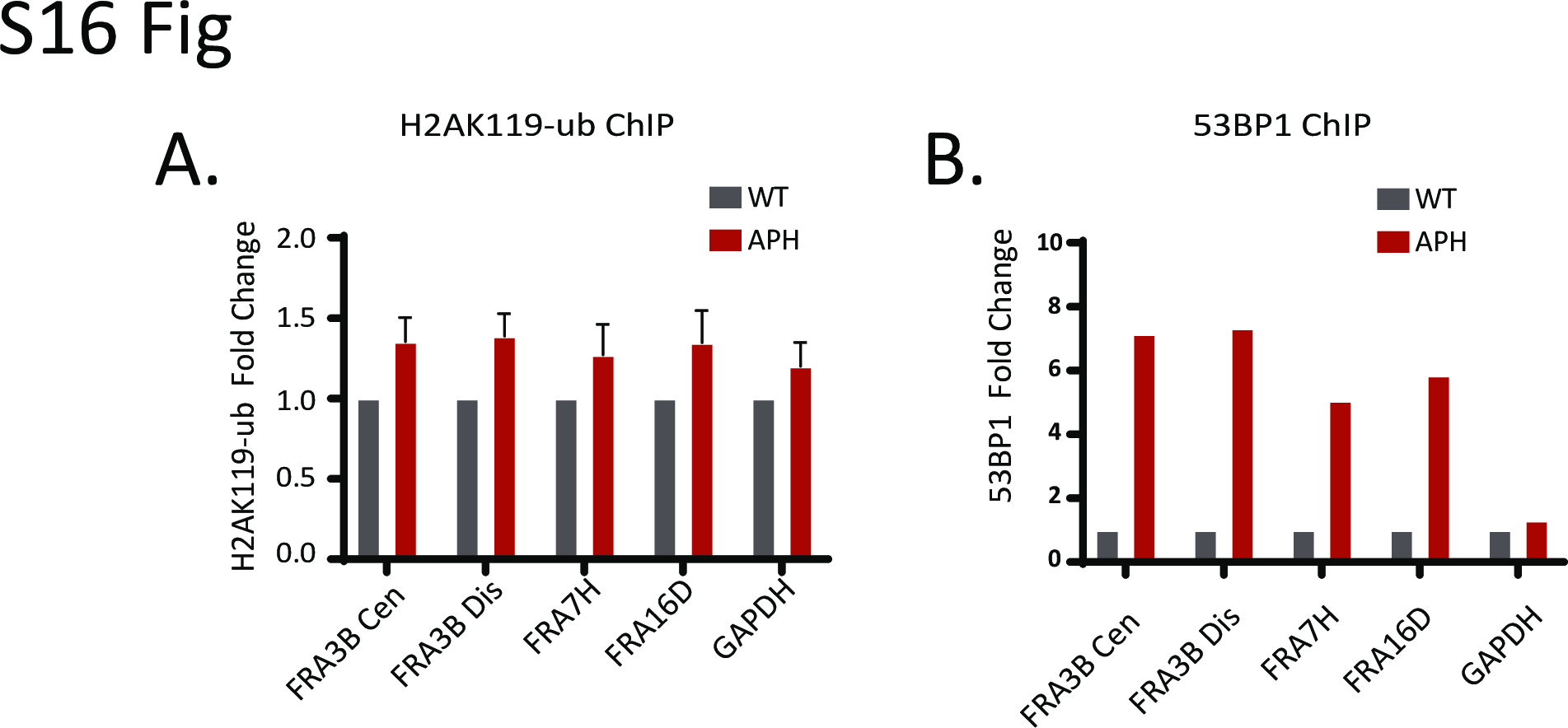

Supplement: S16 Fig — A. qPCR quantification of H2AK119-ub ChIP in T80 cells with or without treatment with 0.4μM Aphidicolin for 16 hours. (N = 4 biological replicates). B. qPCR quantification of anti-53BP1 ChIP in wild type T80 cells with or without treatment with 0.4μM Aphidicolin for 16 hours. This IP was done from one of the same lysates used in A to confirm Aphidicolin activity. (TIF) [file pgen.1008524.s016.tif]
